# Association between concussion and mental health in former collegiate athletes

**DOI:** 10.1186/s40621-014-0028-x

**Published:** 2014-11-17

**Authors:** Zachary Y Kerr, Kelly R Evenson, Wayne D Rosamond, Jason P Mihalik, Kevin M Guskiewicz, Stephen W Marshall

**Affiliations:** 1 Department of Epidemiology, Injury Prevention Research Center, University of North Carolina at Chapel Hill, Bank of America Building, Suite 500, 137 E. Franklin St, Chapel Hill, 27599-7505 NC USA; 2 University of North Carolina at Chapel Hill, Bank of America Building, Suite 306, 137 E. Franklin St, Chapel Hill, NC USA; 3 Department of Exercise and Sport Science, Matthew A. Gfeller Sport-related Traumatic Brain Injury Research Center, 313 Woollen Gymnasium, Chapel Hill, 27599-8605 NC USA; 4 Department of Exercise and Sport Science, Matthew A. Gfeller Sport-related Traumatic Brain Injury Research Center, 2207 Stallings-Evans Sports Medicine Center, Chapel Hill, 27599-8700 NC USA; 5 NCAA Injury Surveillance Program, Datalys Center for Sports Injury Research and Prevention, 401 West Michigan Street, Suite 500, Indianapolis, 46202 IN USA

**Keywords:** Injury, Epidemiology, Traumatic brain injury, Depression, Impulsivity, Aggression

## Abstract

**Background:**

The existing research on the association between concussion and mental health outcomes is largely limited to former professional athletes. This cross-sectional study estimated the association between recurrent concussion and depression, impulsivity, and aggression in former collegiate athletes.

**Methods:**

Former collegiate athletes who played between 1987–2012 at a Division I university completed an online questionnaire. The main exposure, total number of self-recalled concussions (sport-related and non-sport-related), were categorized as: zero (referent), one, two, or three or more concussions. The main outcomes were the depression module of The Patient Health Questionnaire (PHQ-9), the Short Form of the Barratt Impulsiveness scale (BIS15); and the 12-item Short Form of the Buss-Perry Aggression Questionnaire (BPAQ-SF). Depression was categorized into a binomial severity classification that differentiated between no or mild depression (PHQ-9 scores <10) and moderate to severe depression (PHQ-9 scores ≥10). Impulsivity and aggression were kept as continuous outcomes. Binomial regression estimated adjusted prevalence ratios (PR). Linear regression estimated adjusted mean differences (MD).

**Results:**

Of the 797 respondents with complete data (21.9% completion rate), 38.8% reported at least one concussion. Controlling for alcohol dependence and family history of depression, the prevalence of moderate to severe depression among former collegiate athletes reporting three or more concussions in total was 2.4 times that of those reporting zero concussions [95% Confidence Interval (CI): 1.0, 5.7]. Controlling for alcohol dependence, family history of anxiety, relationship status, obtaining a post-graduate degree, and playing primary college sport professionally, former collegiate athletes reporting two or more concussions in total had higher mean scores for impulsivity, compared to those reporting no concussions (2 concussions MD = 2.7; 95% CI: 1.2, 4.1; 3+ concussions MD = 1.9; 95% CI: 0.6, 3.2). Controlling for alcohol dependence, sex, and relationship status, former collegiate athletes reporting three or more concussions in total had a higher mean score for aggression, compared to those reporting no concussions (MD = 3.0; 95% CI: 1.4, 4.7).

**Conclusions:**

Our study found an association between former concussion and greater risk of severe depression and higher levels of impulsivity and aggression among former collegiate athletes. Additional prospective studies better addressing causality and ascertaining valid lifetime concussion histories and medical histories are needed.

## Background

The Centers for Disease Control and Prevention estimates that each year, up to 3.8 million sport-related concussions occur in the United States (US) (Langlois et al. [2006]). Sport-related physical activity is responsible for a large proportion of concussions (Sosin et al. [1996]), thereby placing many athletes at risk of concussion and its potential long-term consequences (Marar et al. [2012]; Mueller and Colgate [2011]). Concussion rates are higher in sports permitting body contact such as football, soccer, hockey, and lacrosse than in low/non-contact sports such volleyball, and swimming (Gessel et al. [2007]; Marar et al. [2012]; Schulz et al. [2004]).

Recurrent concussions are of particular concern, given findings from living retired athletes that suggest that recurrent concussion may also accelerate long-term negative mental health outcomes, particularly depression, mild cognitive impairment, and Alzheimer’s disease (De Beaumont et al. [2009]; DeKosky et al. [2010]; Didehbani et al. [2013]; Guskiewicz et al. [2005], [2007]; Kerr et al. [2012b]). More recently, research has also examined head trauma’s association with chronic traumatic encephalopathy (CTE), a progressive neurodegenerative disorder predominantly observed in professional athletes in high contact sports (e.g., football, boxing, ice hockey, professional wrestling) that have sustained repetitive head trauma (Gavett [2011]; Geddes et al. [1999]; McKee et al., [2010]; Omalu et al [2010]). It has been suggested that CTE may be associated with symptoms of mental health disorders such as depression, impulsivity, and aggression (McKee et al., [2010], [2013]).

However, the existing research is largely limited to samples of former professional athletes and has a number of methodological concerns. First, these studies typically utilized male-only samples that had played collision sports such as football and ice hockey (De Beaumont et al. [2009]; DeKosky et al. [2010]; Didehbani et al. [2013]; Guskiewicz et al. [2005], [2007]; Kerr et al. [2012b]). Thus, it is difficult to generalize findings to former athletes that are younger, female, and from sports with lower levels of contact. Second, with the exception of a few recent studies (Didehbani et al. [2013]; Schwenk et al. [2007]; Weigand et al. [2013]), prior research has made limited use of validated scales to assess mental health outcomes. Third, in many cases, only concussions sustained during professional careers were considered in analyses (Guskiewicz et al. [2005], [2007]; Kerr et al. [2012b]). However, some of the “non-exposed” athletes (i.e., no professional sport concussions) in previous studies may have sustained concussions in other sport- and non-sport-related settings. Not considering a complete concussion history that includes all sport- and non-sport-related concussions across the lifespan could lead to biased effect estimates. Finally, although depression has been previously studied in relation to recurrent concussion, there has been limited research to date on other mental health outcomes that have been speculatively linked to concussion, such as impulsivity and aggression.

The purpose of this study was to estimate the association between recurrent concussion and current levels of depression, impulsivity, and aggression in a cohort of former collegiate athletes. We also examined how use of a concussion history measure that considered only college and professional sport-related concussions would influence effect estimates, relative to a total concussion history that considered all sport- and non-sport-related concussions.

## Methods

### Study design and recruitment

The study utilized a cross-sectional design. An alumni association of a Division I university in the southern US provided us with the email addresses of 3,657 alumni members that were denoted as former collegiate athletes that had played at least one season of a collegiate sport between 1987 and 2012. These former collegiate athletes received an invitation to complete an online self-administered questionnaire hosted on the Qualtrics survey platform. The invitation did not include information about this study’s specific research question.

The inclusion criteria for eligibility into the study cohort were: played at least one season of a collegiate sport at the host university between 1987 and 2012; aged 18 years or older; had a working email address provided by the university alumni association; and able to read and understand English. Ten respondents informed us that they did not fit the eligibility criteria and were excluded. Reminder emails were sent every other week throughout the three-month data collection window (April to June 2013). The Institutional Review Board at The University of North Carolina at Chapel Hill approved all aspects of this study; all respondents provided informed consent prior to participation.

### Measures

The questionnaire included questions on sports history, concussion history, current physical and mental health, and sociodemographic characteristics. The questionnaire was based on the Retired National Football League (NFL) Players cohort health (Kerr et al. [2012b]). An initial version of the questionnaire was pilot-tested on a group of 12 former student-athletes and athletic trainers providing care to collegiate athletes. Wording of questions was then revised based on their feedback prior to deployment.

### Main exposure: self-reported concussion history

Respondents were provided with a definition of concussion adapted from one utilized in previous research (McCrea et al. [2004]). Concussions were defined as occurring typically, but not necessarily, from a blow to the head, and followed by a variety of symptoms that may include any of the following: headache, dizziness, loss of balance, blurred vision, “seeing stars,” feeling in a fog or slowed down, memory problems, poor concentration, nausea, throwing-up, and loss of consciousness. Respondents were then asked to report the number of concussions that they believed they had sustained during participation in sports, including at the high school, college, and (if applicable) professional levels. In addition, respondents reported the number of non-sport-related concussions (e.g., from a car crash, fall, or violence). We reminded respondents that these non-sport-related concussions may also include any childhood injuries that they had been told about, but may not remember. We stratified total concussion history data into four categories: zero (referent), one, two, and three or more concussions.

### Outcome measures of depression, impulsivity, and aggression

#### Depression

The depression module from the Patient Health Questionnaire (PHQ), a self-administered version of the PRIME-MD diagnostic tool, has been used to screen and diagnose health disorders (Kroenke et al. [2001]). The depression module (PHQ-9) (α = 0.85) consists of the nine criteria, scored from “0” (not at all) to “3” (nearly every day), that were provided by the Diagnostic and Statistical Manual of Mental Disorders, 4th edition (DSM-IV). PHQ-9 scores were categorized into a binomial depression severity classification using standardized cutoffs that differentiated between no or mild depression (PHQ-9 scores <10) and moderate to severe depression (PHQ-9 scores ≥10) (Kroenke et al. [2001]). PHQ-9 is not able to account for those individuals that may score low due to being treated for depression so we excluded those respondents with PHQ-9 scores <10 that reported being currently treated/medicated for depression (n = 28).

#### Impulsivity

The Short Form of the Barratt Impulsiveness scale (BIS15) (α = 0.84) (Spinella [2007]) consists of 15 items answered on a four-point scale (1 = “rarely/never”; 4 = “almost always”). Questions focused on: task-focus (attentional); acting without thinking (motor); and not thinking about the future (non-planning). Because standardized cutoffs for the BIS15 do not exist, scores were kept as continuous outcomes.

#### Aggression

The 12-item Short Form of the Buss-Perry Aggression Questionnaire (BPAQ-SF) (α = 0.89) utilizes a five-point scale (1 = “extremely uncharacteristic of me”; 5 = “extremely characteristic of me”) (Bryant and Smith [2001]). Questions focused on physical aggression, verbal aggression, anger, and hostility. Because standardized cutoffs for the BPAQ-SF do not exist, scores were kept as continuous outcomes.

### Covariates

In order to explore for potential confounding effects from other individual characteristics and behaviors, we collected data on sociodemographics and sports history (Table 1). We also collected data on alcohol dependence using the using the CAGE questionnaire (Ewing [1984]). Last, additional covariates were collected for inclusion for specific models given previous research (Beekman et al. [1995]; Bushman and Cooper [1990]; Haynes et al. [2005]; Kleiber et al. [1987]; Rowland et al. [2002]).

**Table 1:** **Covariates included in models exploring the association between recurrent concussion and mental health outcomes**

Variable	Categories	Included in model		
		Depression	Impulsivity	Aggression
Sociodemographics		x	x	x
Sex	Male/Female	x	x	x
Race/ethnicity	Non-Hispanic White/All other race/ethnicity combinations	x	x	x
Current age	Continuous	x	x	x
Current body mass index	Continuous	x	x	x
Relationship status	Single/In a relationship	x	x	x
Education level (obtained graduate degree)	Yes/No	x	x	x
Work status (currently employed at least part time)	Yes/No	x	x	x
Disability status	Disabled/Non-disabled	x	x	x
Sport history				
Primary college sport played (by level of playing contact)	Collision/High contact/Low/non-contact	x	x	x
Number of years since played college sports	Continuous	x	x	x
Played professional sports	Yes/No	x	x	x
Total number of years played primary sport	Continuous	x	x	x
Career-ending injury	Yes/No	x		
Additional variables				
Alcohol dependence	Continuous	x	x	x
Family history of depression	Yes/No	x		
Family history of anxiety	Yes/No		x	

### Statistical analyses

Each model was run with total concussion history as the main exposure. We then reran models using a concussion history that considered only college and professional sport-related concussions to determine how effect estimates changed relative to a total concussion history. Effect measure modification was assessed between concussion history and covariates. Model building for each outcome are explained below. Level of significance for all analyses was set a priori at *P* < 0.05. Analyses were conducted on SAS 9.3 (SAS Institute Inc.; Cary, NC).

#### Depression models

Crude prevalence ratios (PR) and prevalence differences (PD) were obtained with classical tabular methods. Adjusted PR and 95% confidence intervals (CI) were estimated using binomial regression. Adjusted PD and 95% CI were estimated using linear risk regression (Spiegelman and Hertzmark [2005]). Fitting algorithms for binomial regression and linear risk regression models were stabilized using Poisson residual and robust variance estimation (Greenland [2004]; Spiegelman and Hertzmark [2005]; Zou [2004]). Binomial regression models predicting depression utilized forward selection model building with total concussion history as the main exposure. To ensure consistency, the covariates from this model were used in all additional models.

#### Impulsivity and aggression models

Mean differences (MD) were estimated with linear regression. To satisfy assumptions of linear regression, models originally utilized transformed variables. However, these transformations did not change the direction or the significance of effect estimates. Thus, effect estimates from models with untransformed variables were reported. In addition, we did not find evidence of multicollinearity as no covariates yield variance inflation factors (VIF) above the recommended cut-off point of 10 (Hair et al. [1995]). Linear regression models predicting impulsivity and aggression utilized backward selection model building with total concussion history as the main exposure. To ensure consistency, the covariates from this model were used in all additional models.

## Results

### Sample characteristics

We received complete data from 797 (21.9%) former collegiate athletes. Respondents from 27 different collegiate sports were included, with a majority playing in men’s football, followed by women’s rowing, men’s and women’s fencing, and women’s track and field (Table 2). The sport distribution was similar to the to the 2013/14 athlete roster at the host university that our sample had attended. The mean time since respondents’ last year of participating in collegiate sport was 14.5 years [Standard Deviation (SD) = 7.4], with 29.4% playing within the past ten years ago (Table 3). On average, respondents played their last year of collegiate sports slightly longer ago than non-respondents (*P* < 0.001). The mean time of respondents’ playing their primary sport was 10.7 years (SD = 5.7) with 55.5% playing for at least ten years. Among respondents, 70.2% were in a relationship; 87.7% were currently employed at least part-time; 11.5% played their primary sport professionally; 16.2% had sustained a career-ending injury; and 0.8% were on disability.

**Table 2:** **Distributions of former collegiate athlete cohort and 2013/14 school year athlete roster from host institution, by sport**

Sport	Former collegiate athlete cohort		2013/14 roster	
	n	%	N	%
Men’s Baseball	31	3.9	34	4.4
Men’s Basketball	22	2.8	16	2.1
Men’s Cross Country	8	1.0	20	2.6
Men’s Fencing	54	6.8	28	3.6
Men’s Football	75	9.4	122	15.8
Men’s Lacrosse	35	4.4	43	5.6
Men’s Soccer	22	2.8	31	4.0
Men’s Swimming and Diving	44	5.4	32	4.2
Men’s Tennis	13	1.6	14	1.8
Men’s Track and Field	34	4.3	48	6.2
Men’s Wrestling	27	3.4	35	4.5
Women’s Basketball	14	1.8	13	1.7
Women’s Cross Country	14	1.8	15	1.9
Women’s Fencing	48	6.0	19	2.5
Women’s Field Hockey	29	3.6	25	3.2
Women’s Golf	16	2.0	8	1.0
Women’s Gymnastics	21	2.6	13	1.7
Women’s Lacrosse	25	3.1	33	4.3
Women’s Rowing	66	8.3	33	4.3
Women’s Soccer	30	3.8	36	4.7
Women’s Softball	30	3.8	22	2.9
Women’s Swimming and Diving	45	5.6	33	4.3
Women’s Tennis	11	1.4	8	1.0
Women’s Track and Field	52	6.5	38	4.9
Women’s Volleyball	27	3.4	18	2.3
Cheerleading	3	0.4	15	1.9
Equestrian^a^	1	0.1	19	2.5
**Total**	**797**	**100.0**	**771**	**100.0**

NOTE: Host institution statistics originated from their athletics website.

^a^Equestrian considered a club sport.

**Table 3:** **Sociodemographics and sports history of former collegiate athlete cohort (n = 797)**

Sociodemographics/sports history	n	%	Sociodemographics/sports history	n	%
Sex			Highest education level		
Male	376	47.2	High school/GED	6	0.7
Female	421	52.8	Bachelor’s degree	377	47.4
			Post-graduate degree	413	51.9
Age (in years)			Missing	1	
Less than 29	212	26.6			
30 to 34	139	17.4	Total years played sport		
35 to 39	158	19.8	Less than 5	147	18.5
40 to 44	167	21.0	5 to 9	206	25.9
45 and over	121	15.2	10 to 14	221	27.8
			15 to 19	188	23.7
Race/ethnicity			20 to 24	25	3.1
Non-Hispanic White	686	86.1	25 or more	7	0.9
Non-Hispanic Black	71	8.9	Missing	3	
Non-Hispanic Asian/PI^a^	11	1.4			
Mixed race	29	3.6	Years since playing college sports		
			Less than 5	78	10.0
Current body mass index^b^			5 to 9	152	19.4
Underweight/Normal	427	55.4	10 to 14	170	21.7
Overweight	264	33.2	15 to 19	143	18.3
Obese	90	11.3	20 to 24	149	19.1
Missing	3		25 or more	90	11.5
			Missing	15	

^a^PI = Pacific Islander.

^b^WHO classifications: Underweight/Normal (<25.0 kg/m^2^); Overweight (25.0 – 29.9 kg/m^2^); Obese (≥30.0 kg/m^2^).

### Concussion history

Concussions (sport- and non-sport-related) were reported by 307 (38.8%) respondents (Table 4). Five respondents did not provide information on non-sport-related concussions and were excluded from analyses. Among those reporting no concussions during collegiate and professional sports (n = 684), 28.6% reported sustaining at least one concussion elsewhere (e.g., high school sports, non-sport-related activities). Agreement between total concussions and concussions sustained during college and professional sport-related was moderate (weighted Cohen’s Kappa = 0.41; 95% CI: 0.35, 0.47).

**Table 4:** **Distributions and mean values of mental health outcomes, by self-reported total concussion history**

Mental health outcome	Number of total concussions ^a^				Total
	0 (n = 485)	1 (n = 145)	2 (n = 68)	3+ (n = 94)	
Depression (PHQ-9)^b^					
Mean (SD)	2.5 (3.0)	2.9 (3.6)	4.5 (4.6)	4.5 (5.0)	3.0 (3.7)
Categories, n (%)					
Minimal (0–4)	370 (79.9)	114 (80.3)	39 (58.2)	57 (63.3)	580 (76.1)
Mild (5–9)	80 (17.3)	20 (14.1)	21 (31.3)	25 (27.8)	146 (19.2)
Moderate (10–14)	9 (2.0)	5 (3.5)	4 (6.0)	4 (4.4)	22 (2.9)
Moderately Severe (15–19)	3 (0.7)	3 (2.0)	1 (1.5)	1 (1.1)	8 (1.0)
Severe (20–27)	1 (0.2)	0 (0.0)	2 (3.0)	3 (3.3)	6 (0.8)
% ≥10^c^	2.8%	5.6%	10.4%	8.9%	4.7%
.					
Impulsivity (BIS15)					
Mean (SD)	25.1 (5.2)	27.0 (6.4)	28.4 (7.0)	27.7 (7.4)	26.0 (6.0)
Subscale Mean (SD)					
Attentional	8.5 (2.4)	9.4 (2.7)	10.3 (3.0)	9.9 (3.3)	9.0 (2.7)
Motor	8.1 (2.1)	8.7 (2.5)	9.0 (2.6)	8.6 (2.5)	8.4 (2.3)
Non-planning	8.4 (2.6)	9.0 (2.8)	9.1 (2.8)	9.2 (3.2)	8.7 (2.7)
.					
Aggression (BPAQ-SF)					
Mean (SD)	18.0 (6.3)	18.8 (7.8)	20.6 (7.7)	22.4 (10.0)	18.9 (7.4)
Subscale Mean (SD)					
Physical aggression	3.5 (1.3)	3.8 (1.9)	3.9 (1.7)	4.5 (2.7)	3.7 (1.7)
Verbal aggression	5.3 (2.4)	5.4 (2.5)	6.2 (2.7)	6.6 (3.1)	5.5 (2.6)
Anger	4.7 (2.2)	5.1 (2.7)	5.5 (2.5)	5.7 (2.9)	5.0 (2.5)
Hostility	4.5 (2.2)	4.6 (2.5)	5.0 (2.7)	5.6 (3.3)	4.7 (2.5)

NOTE: PHQ-9 = Patient Health Questionnaire for Depression; BIS15 = Short Form of the Barratt Impulsiveness scale; BPAQ-SF = 12-item Short Form of the Buss-Perry Aggression Questionnaire; SD = Standard deviation; Sum of number of concussions may not equal 797 due to missing data for non-sport-related concussions and mental health outcomes.

^a^Total concussions includes all sport- and non-sport-related concussions.

^b^Excludes respondents with PHQ-9 scores <10 that reported being currently treated/medicated for depression (n = 28).

^c^PHQ-9 score ≥10 indicates moderate to severe depression.

### Mental health outcomes

Among respondents, 4.7% had moderate to severe depression (Table 4). Average BIS15 scores for impulsivity were 26.0 (SD = 6.0). The BPAQ-SF subscale scores for aggression (overall score mean: 18.7, SD = 7.4) varied, with the highest score being verbal aggression (5.5, SD = 2.6) and the lowest score being physical aggression (3.7, SD = 1.7).

### Concussion and mental health outcome models

#### Depression

Crude associations were observed between recurrent concussion and depression (Table 5). In multivariate binomial regression models controlling for covariates, the association was attenuated but still present. Controlling for alcohol dependence and family history of depression, the prevalence of moderate to severe depression among former collegiate athletes reporting three or more concussions in total was 2.4 times that of former collegiate athletes reporting zero concussions (95% CI: 1.0, 5.7).

**Table 5:** **Prevalence ratios and prevalence differences of depression, by self-reported total concussion history** ^**a**^

Total concussions	n	Crude PR		Adjusted PR		Crude PD		Adjusted PD	
		PR	95% CI	PR	95% CI	PD	95% CI	PD	95% CI
Depression (PHQ-9)^b^									
0	463	1		1		0		0	
1	142	2.0	(0.8, 4.7)	1.6	(0.7, 3.7)	0.03	(-0.01, 0.07)	0.00	(-0.03, 0.03)
2	67	3.7	(1.5, 9.0)	2.4	(0.9, 6.3)	0.07	(0.00, 0.15)	0.03	(-0.02, 0.09)
3+	90	3.2	(1.4, 7.4)	2.4	(1.0, 5.7)	0.06	(0.00, 0.12)	0.04	(-0.01, 0.09)

NOTE: PHQ-9 = Patient Health Questionnaire for Depression; PR = Prevalence ratio; PD = Prevalence difference; CI = Confidence interval.

^a^Total concussions includes all sport- and non-sport-related concussions.

^b^Excludes respondents with PHQ-9 scores <10 that reported being currently treated/medicated for depression (n = 28);

models utilize split where 0 = <10 on PHQ-9, 1 = ≥10 on PHQ-9 (score ≥10 indicates moderate to severe depression). Models predicting PRs utilized binomial regression. Models predicting PDs utilized linear regression; adjusted models control for alcohol dependence and family history of depression.

#### Impulsivity

Modest crude associations were observed between recurrent concussion and impulsivity (Table 6). However, estimates were attenuated following adjustment for covariates. Controlling for covariates (alcohol dependence, family history of anxiety, relationship status, obtaining a post-graduate degree, played primary college sport professionally), former collegiate athletes reporting two concussions in total had higher mean scores for impulsivity, compared to those reporting no concussions (MD = 2.7; 95% CI: 1.2, 4.1). Former collegiate athletes reporting three or more concussions in total also had higher mean scores for impulsivity (MD = 1.9; 95% CI: 0.6, 3.2), although this association was slightly weaker than that for two concussions.

**Table 6:** **Mean differences of impulsivity and aggression, by self-reported total concussion history** ^**a**^

Total concussions	n	Crude MD		Adjusted MD	
		MD	95% CI	MD	95% CI
Impulsivity (BIS15)^b, c^					
0	476	0		0	
1	142	2.0	(0.9, 3.1)	1.1	(-0.0, 2.1)
2	67	3.4	(1.8, 4.9)	2.7	(1.2, 4.1)
3+	92	2.7	(1.4, 4.0)	1.9	(0.6, 3.2)
Aggression (BPAQ-SF)^b, d^					
0	477	0		0	
1	143	0.8	(-0.5, 2.2)	0.0	(-1.3, 1.4)
2	67	2.7	(0.8, 4.5)	1.8	(-0.0, 3.7)
3+	94	4.5	(2.9, 6.1)	3.0	(1.4, 4.7)

NOTE: BIS15 = Short Form of the Barratt Impulsiveness scale; BPAQ-SF = 12-item Short Form of the Buss-Perry Aggression Questionnaire; MD = Mean difference; CI = Confidence interval.

^a^Total concussions includes all sport- and non-sport-related concussions.

^b^Models utilized continuous outcomes and predicted mean differences using linear regression.

^c^Adjusted models control for alcohol dependence, family history of anxiety, relationship status, education (obtained post-graduate degree), played primary college sport professionally.

^d^Adjusted models control for alcohol dependence, sex, relationship status , played primary college sport professionally, and current BMI.

#### Aggression

Crude associations were observed between recurrent concussion and aggression (Table 6), but were attenuated following adjustment for covariates. Controlling for alcohol dependence, sex, and relationship status, former collegiate athletes reporting three or more concussions in total had a higher mean score for aggression, compared to those reporting no concussions (MD = 3.0; 95% CI: 1.4, 4.7).

### Models using only college and professional sport-related concussions

We repeated analyses with a concussion history that considered only college and professional sport-related concussions. Effect estimates tended to vary from those obtained utilizing total concussion history (Tables 7 & 8). In addition, due to the lower cell sizes for individuals sustaining college and professional sport-related concussions, effect estimates were less precise.

**Table 7:** **Prevalence ratios and prevalence differences of depression, by self-reported college and professional sport concussion history**

Number of concussions ^a^	n	Crude PR		Adjusted PR		Crude PD		Adjusted PD	
		PR	95% CI	PR	95% CI	PD	95% CI	PD	95% CI
Depression (PHQ-9)^b^									
0	655	1		1		0		0	
1	65	0.7	(0.2, 2.8)	0.6	(0.2, 2.5)	-0.01	(-0.06, 0.03)	-0.07	(-0.06, 0.03)
2	21	1.1	(0.2, 7.5)	1.2	(0.2, 9.5)	0.00	(0.09, 0.10)	0.10	(-0.06, 0.13)
3+	26	3.5	(1.3, 9.2)	3.4	(1.5, 7.5)	0.11	(-0.03, 0.25)	0.04	(-0.08, 0.20)

NOTE: PHQ-9 = Patient Health Questionnaire for Depression; PR = Prevalence ratio; PD = Prevalence difference; CI = Confidence interval.

^a^Total concussions includes all concussions sustained during participation in college and professional sports.

^b^Excludes respondents with PHQ-9 scores <10 that reported being currently treated/medicated for depression (n = 28);

models utilize split where 0 = <10 on PHQ-9, 1 = ≥10 on PHQ-9 (score ≥10 indicates moderate to severe depression). Models predicting PRs utilized binomial regression. Models predicting PDs utilized linear regression; adjusted models control for alcohol dependence and family history of depression.

**Table 8:** **Mean differences of impulsivity and aggression, by self-reported college and professional sport concussion history**

Total concussions	n	Crude MD		Adjusted MD	
		MD	95% CI	MD	95% CI
Impulsivity (BIS15)^a, b^					
0	669	0		0	
1	65	1.7	(0.2, 3.2)	0.8	(-0.7, 2.3)
2	21	2.2	(-0.4, 4.9)	1.7	(-0.9, 4.3)
3+	27	1.8	(-0.5, 4.1)	1.5	(-0.7, 3.7)
Aggression (BPAQ-SF)^a, c^					
0	672	0		0	
1	66	1.5	(-0.4, 3.3)	0.4	(-1.4, 2.2)
2	21	3.9	(0.7, 7.1)	2.5	(-0.8, 5.7)
3+	27	3.3	(0.5, 6.2)	2.1	(-0.7, 4.9)

NOTE: BIS15 = Short Form of the Barratt Impulsiveness scale; BPAQ-SF = 12-item Short Form of the Buss-Perry Aggression Questionnaire; MD = Mean difference; CI = Confidence interval.

^a^Models utilized continuous outcomes and predicted mean differences using linear regression.

^b^Adjusted models control for alcohol dependence, family history of anxiety, relationship status, education (obtained post-graduate degree), played primary college sport professionally.

^c^Adjusted models control for alcohol dependence, sex, relationship status , played primary college sport professionally, and current BMI.

## Discussion

This study extends previous studies that examined the association between recurrent concussion and mental health (De Beaumont et al. [2009]; DeKosky et al. [2010]; Didehbani et al. [2013]; Guskiewicz et al. [2005], [2007]; Kerr et al. [2012b]) by exploring impulsivity and aggression as outcomes. This is also the first study to examine these associations in a cohort of former collegiate (rather than professional) athletes, most of whom had not played professionally. The study cohort is the most diverse sample studied to date, drawing from a diverse range of collegiate sports that included males and females, some of whom played sports with little or no body contact, but still were at risk of sustaining head injuries.

Our cohort of former collegiate athletes had a lower prevalence of moderate to severe depression, compared to a sample of former NFL players (4.7% vs. 14.7%) (Schwenk et al. [2007]). Nevertheless, the findings contribute to a growing body of evidence that links the risk of depression to self-reported concussion history in former athletes. Cross-sectional data (Guskiewicz et al. [2007]) indicated that compared to retired NFL players that reported zero concussions during their professional football career, those reporting three or more concussions were three times as likely (95% CI: 2.3, 4.1) to report that they were diagnosed with depression. A follow-up study (Kerr et al. [2012b]), which incorporated longitudinal data and examined incidence of depression, found larger effect estimates and a stronger dose-response relationship between concussion history and the nine-year risk of depression diagnosis. A recent case-control study (Didehbani et al. [2013]) also found a strong association between the number of lifetime concussions and depressive symptom severity, particularly cognitive symptoms such as feelings of sadness, guilt, and critical self-evaluation.

Compared to former collegiate athletes reporting sustaining zero concussions, former collegiate athletes reporting three or more concussions had higher average aggression and impulsivity scores. However, we caution interpretation of these results. There are currently no validated scales that allow for retrospective recall of these outcomes. Therefore, we had no means of assessing whether aggression (and impulsivity) were manifested in participants prior to the onset of concussion. Subjects with high levels of aggression in this study may have had the same aggression levels prior to their concussions, and outcomes such as aggression and impulsivity may have influenced the probability of exposure (e.g., aggressive playing behavior may have increased the risk of concussion). We were unable to distinguish athletes properly treated for depression, impulsivity, and aggression (and thus scoring low on our scale measures), and those athletes who scored low on our scale measures because they were without these conditions. To manage this with the PHQ-9, we excluded those respondents with no to mild depression, but reported being treated/medicated for depression. We were unable to impose the same restriction for impulsivity and aggression. At the same time, we are unsure whether the mean differences in impulsivity and aggression were clinically relevant. Future longitudinal studies that examine athlete cohorts and mental health outcomes longitudinally, while incorporating treatment/medication as time-varying covariates, will provide stronger assessments of these causal relationships.

Our main exposure, concussion history, may be prone to measurement and recall bias (Kerr et al. [2012a]). However, self-reported concussion history is easier to obtain than medical reports and also may be more complete. Recent findings have suggested a history of underreporting of concussion in clinical records, with between 35% and 62% of athletes not reporting all sustained concussions to coaches and/or team medical staff (Broglio et al. [2010]; Kroshus et al. [2014]; Llewellyn et al. [2014]; McCrea et al. [2004]; Register-Mihalik et al. [2013]). Our main exposure also accounts for the number of concussions sustained, but is unable to account for variations in the time since injury and the time between multiple concussions.

We also caution that concussion effects for some mental health outcomes, such as impulsivity, may not be apparent in our cohort due to their young age, particularly in comparison to the Retired NFL Players Cohort (Guskiewicz et al. [2005], [2007]; Kerr et al. [2012b]), and the relatively short time (mean of 14.5 years) since they played collegiate sport. Adverse outcomes may take some time to develop and occur at a later age. Continued research with former athletes of all ages will help determine a more precise age range at which the onset of negative mental health outcomes occurs.

Last, our findings illustrated that when solely considering college and professional sport-related concussions, as opposed to all sports- and non-sport-related concussions, effect estimates became less precise and in some cases, changed considerably. Previous research on retired professional football players (Guskiewicz et al. [2005], [2007]; Kerr et al. [2012a], [b]) opted to utilize professional sport-related concussion history for multiple reasons. First, former athletes probably have better recall of these concussions than earlier concussions due to memory decay effects. In addition, on-site clinical coverage during college and professional sports may have led to better detection and diagnoses of these concussions, as compared to those sustained prior. Last, moderate correlation was found between the reported number of concussions sustained during former NFL players’ professional and collegiate careers (Guskiewicz et al. [2003]). However, in this cohort of former collegiate athletes that included sports with low levels of contact, 28.6% of those reporting no concussions during collegiate and professional sports had sustained at least one concussion elsewhere (e.g., high school sports, non-sport-related activities). A complete concussion history may provide more valid estimates of the effects of sustaining concussions than a sport-related concussion history.

### Limitations

Although repeated efforts were made to contact our target sample of 3,657 former collegiate athletes, our completion rate among eligible respondents was low (21.9%). The sample originated from one university and was restricted to those that had played at least one season in 1987–2012. Thus, our findings may not be generalizable to non-respondents, former athletes not in contact with the university alumni association, and former athletes from other universities, playing eras, or playing levels. Our sample also represents a small proportion of all former collegiate athletes in the US. Nonetheless, this study provides estimates from a diverse population of former athletes. The study was cross-sectional, although findings highlight the need for longitudinal examinations of former athletes, particularly those that incorporate treatment/medication as time-varying covariates. Future studies should also consider additional covariates that capture a more in-depth history of medical issues, education problems, and/or aggression/arrests. As previously mentioned, information bias related to the exposure and outcomes may have resulted in biased effect estimates. Last, differential recall bias could also result from former players experiencing normal cognitive decay due to aging, which may prompt them to dwell more on their health and as a result, spuriously result in increased attribution of life changes to concussions. In addition, concussion and its care have evolved significantly over the 25-year span of our cohort. As a result, more recent alumni may have worked with sports medical staff that are better educated and more skilled at the detection and management of concussions, thus helping their recall of such injuries.

## Conclusions

Our study found an association between former concussion and greater risk of severe depression and higher levels of impulsivity and aggression among collegiate athletes. However, additional prospective studies that better address causality are needed. In particular, studies should better ascertain a valid lifetime concussion history, as well as medical histories regarding diagnosis, treatment, and management of mental health issues. Nevertheless, improved access to mental health care will benefit former collegiate athletes as well as the general population.

## Abbreviations

BIS15: Short Form of the Barratt Impulsiveness scale
BPAQ-SF: 12-item Short Form of the Buss-Perry Aggression Questionnaire
CI: Confidence interval
CTE: Chronic traumatic encephalopathy
MD: Mean difference
PD: Prevalence difference
PHQ-9: Depression module of The Patient Health Questionnaire
PR: Prevalence ratio
